# Acetate Availability and Utilization Supports the Growth of Mutant Sub-Populations on Aging Bacterial Colonies

**DOI:** 10.1371/journal.pone.0109255

**Published:** 2014-10-02

**Authors:** Jessica M. Bergman, Marie Wrande, Diarmaid Hughes

**Affiliations:** Department of Medical Biochemistry and Microbiology, Biomedical Center, Uppsala University, Uppsala, Sweden; University of Massachusetts Medical School, United States of America

## Abstract

When bacterial colonies age most cells enter a stationary phase, but sub-populations of mutant bacteria can continue to grow and accumulate. These sub-populations include bacteria with mutations in *rpoB* (RNA polymerase β-subunit) or *rpoS* (RNA polymerase stress-response sigma factor). Here we have identified acetate as a nutrient present in the aging colonies that is utilized by these mutant subpopulations to support their continued growth. Proteome analysis of aging colonies showed that several proteins involved in acetate conversion and utilization were upregulated during aging. Acetate is known to be excreted during the exponential growth phase but can be imported later during the transition to stationary phase and converted to acetyl-CoA. Acetyl-CoA is used in multiple processes, including feeding into the TCA cycle, generating ATP via the glyoxylate shunt, as a source of acetyl groups for protein modification, and to support fatty acid biosynthesis. We showed that deletion of *acs* (encodes acetyl-CoA synthetase; converts acetate into acetyl-CoA) significantly reduced the accumulation of *rpoB* and *rpoS* mutant subpopulations on aging colonies. Measurement of radioactive acetate uptake showed that the rate of conversion decreased in aging wild-type colonies, was maintained at a constant level in the *rpoB* mutant, and significantly increased in the aging *rpoS* mutant. Finally, we showed that the growth of subpopulations on aging colonies was greatly enhanced if the aging colony itself was unable to utilize acetate, leaving more acetate available for mutant subpopulations to use. Accordingly, the data show that the accumulation of subpopulations of *rpoB* and *rpoS* mutants on aging colonies is supported by the availability in the aging colony of acetate, and by the ability of the subpopulation cells to convert the acetate to acetyl-CoA.

## Introduction

It was previously shown that mutants resistant to rifampicin, carrying mutations in *rpoB* (encoding the β-subunit of RNA polymerase) accumulate in aging colonies of *Salmonella* Typhimurium and *Escherichia coli* [1], [2], [3], [4]. This accumulation occurs because many different rifampicin-resistant (Rif^R^) mutants continue to grow after wild-type cells in the colony enter stationary phase [4]. The ability of mutants to postpone their entry into stationary phase is of general interest because natural environments are often nutrient-poor and bacteria can spend much of their time in a non-growth state, punctuated by periods of rapid growth when nutrient becomes available. Accordingly, mutants that can extend the length of their growth phase beyond the average may gain an advantage because they will increase as a proportion of the population prior to the next period of nutrient availability and rapid growth. Mutations in *rpoS*, encoding the general stress response sigma factor RpoS, have previously been shown to increase bacterial survival in long-term liquid and colony stationary phase [5], [6], [7], [8].

In this manuscript we confirm that *rpoS* mutants also accumulate in stationary phase colonies. We also asked whether we could identify a specific nutrient that was important to support the continued growth of mutant subpopulations of cells in or on stationary phase bacterial colonies. We report here that the availability of acetate in aging colonies, and the ability of subpopulations to convert that acetate into acetyl-CoA, are critically important to support the growth of *rpoB* and *rpoS* mutants on wild-type stationary phase colonies.

## Results and Discussion

### Rif^R^ and RpoS mutants each accumulate as colonies age

We have previously shown that Rif^R^ mutants grow and accumulate in aging wild-type colonies of *S.* Typhimurium [4]. RpoS mutants have been shown by others to have a growth advantage (GASP phenotype), and to accumulate, in aging liquid cultures of *E. coli* [5], [6]. We asked whether RpoS mutants would be selected in aging colonies of *Salmonella* and whether RpoS mutants would have a growth advantage if added onto aging colonies. Colonies of wild-type *S.* Typhimurium [9] were aged for 15 weeks on LB agar (LA) in sealed plastic bags. Sequence analysis revealed that a high proportion of the surviving bacteria (3/18 independent clones tested) carried mutations in *rpoS*. These RpoS mutants, including one with an in-frame stop codon, each had a growth advantage over the wild-type when spotted onto aging wild-type colonies (Table S1).

**Figure 1:**
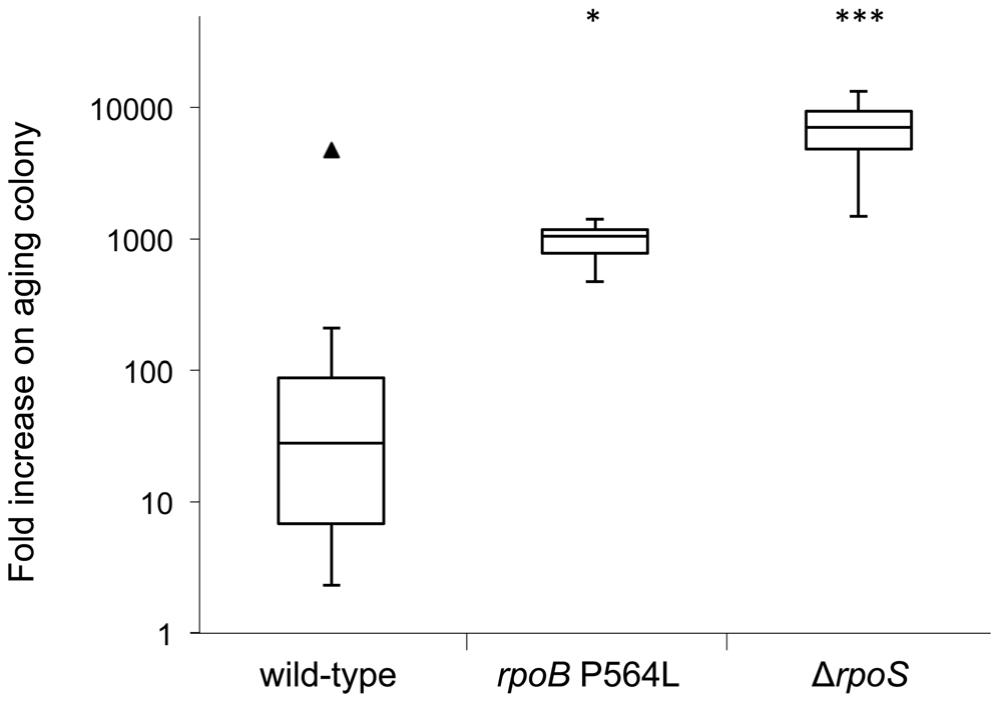
RpoB and RpoS mutants have a growth advantage on aging colonies. Fold increase in wild-type and mutant cells added to 24 h wild-type colonies and allowed to age for a further 7 days. The box plots show the first quartile, median, and third quartile values. Outlier indicated by a triangle. Statistical significance of differences in the distribution of values between strains, compared to the wild-type, is given in Table S2 and indicated in the figure by asterisks (*  = 95% confidence interval, ***  = 99.9% confidence interval).

To confirm that loss of RpoS activity conferred a growth advantage when mutant bacteria were added onto an aging colony, we constructed a precise deletion of *rpoS* in *S.* Typhimurium 14028s by λ-Red recombineering [10], [11]. We then measured and compared the relative ability of the wild-type and of two isogenic mutant strains (Δ*rpoS* and Rif^R^ *rpoB* P564L) to grow and accumulate when added as a subpopulation onto 24 h old wild-type colonies which were then allowed to age for a further 7 days. Each of the two mutants had a significant growth advantage relative to the wild-type on the aging colonies (Figure 1, Table S2). We concluded that the mutations, Δ*rpoS* and *rpoB* P564L, facilitate continued bacterial growth on aging colonies. These data are in agreement with a study of decades-old agar stabs of *S.* Typhimurium, where it was found that surviving cells in 10 of 27 examined vials had a mutated *rpoS* gene [12]. This suggests that loss or change of function of RpoS can confer an advantage in aging environments.

### Carbon metabolism proteins are upregulated in aging colonies

We hypothesized that as bacterial colonies age the bacteria within them would upregulate proteins that were important to support the transition into stationary phase, and/or support continued growth. To gain insight into these phenotypes we made a shotgun proteome analysis of total protein prepared from 1 day-old and 7 day-old colonies of the wild-type, and of the isogenic *rpoB* P564L and Δ*rpoS* mutants [13]. Over 630 different proteins were identified in each of these samples (Table S3). The data were examined for proteins that were strongly upregulated during colony aging, and also present at a relatively high level in 7 day-old colonies. Many of the proteins fitting this profile are annotated as being involved in carbon utilization or carbon scavenging. Notably, these included aconitate hydratase (AcnA), identified as the single most abundant protein, and the most strongly upregulated protein, in 7 day-old colonies of the *rpoB* mutant strain. AcnA was also highly abundant and strongly upregulated in 7 day-old colonies of the wild-type and the RpoS mutant. In contrast, the level of AcnA in preparations from day 1 colonies of all three strains was very low. AcnA plays an important enzymatic role in the TCA cycle by being responsible for the isomerization of citrate and iso-citrate via cis-aconitate. It was also notable that four enzymes, Acs, AcnA, AceA, and AceB, that operate consecutively in the pathway of acetate utilization were upregulated in all three strains and present at relatively high levels in preparations from 7 day-old colonies (Table S3). In addition, GltA (citrate synthetase; required for catalyzing the interaction of oxaloacetate with acetyl-CoA to make citrate) was upregulated and present at a high level in 7 day-old colonies of all three genotypes studied. Transcriptome data from *E. coli* is in broad agreement with this proteome data and shows upregulation of mRNA for *acnA*, *aceA*, *aceB* and *gltA* in wild-type and an *rpoS* mutant aged for 7 days [8], [14].

Taken together, the data from the shotgun proteome analysis suggested that in aging colonies, bacteria upregulated genes required for the utilization of acetate as a carbon source. The potential significance of this is that bacteria growing exponentially on good carbon sources produce and excrete acetate when the inflow of carbon is in excess of what can be processed with respect to central metabolic pathways [15], [16], [17], [18]. During the transition to stationary phase, when the preferred carbon source has been consumed, bacteria then undergo a metabolic switch and import and consume the acetate that had previously been excreted during exponential growth [19]. Because the shotgun proteome analysis is not strictly quantitative an additional quantitative protein analysis was made to accurately determine the relative quantities of several key proteins involved in acetate utilization in colonies of wild-type and mutant strains.

### Quantitative proteome analysis

**Figure 2:**
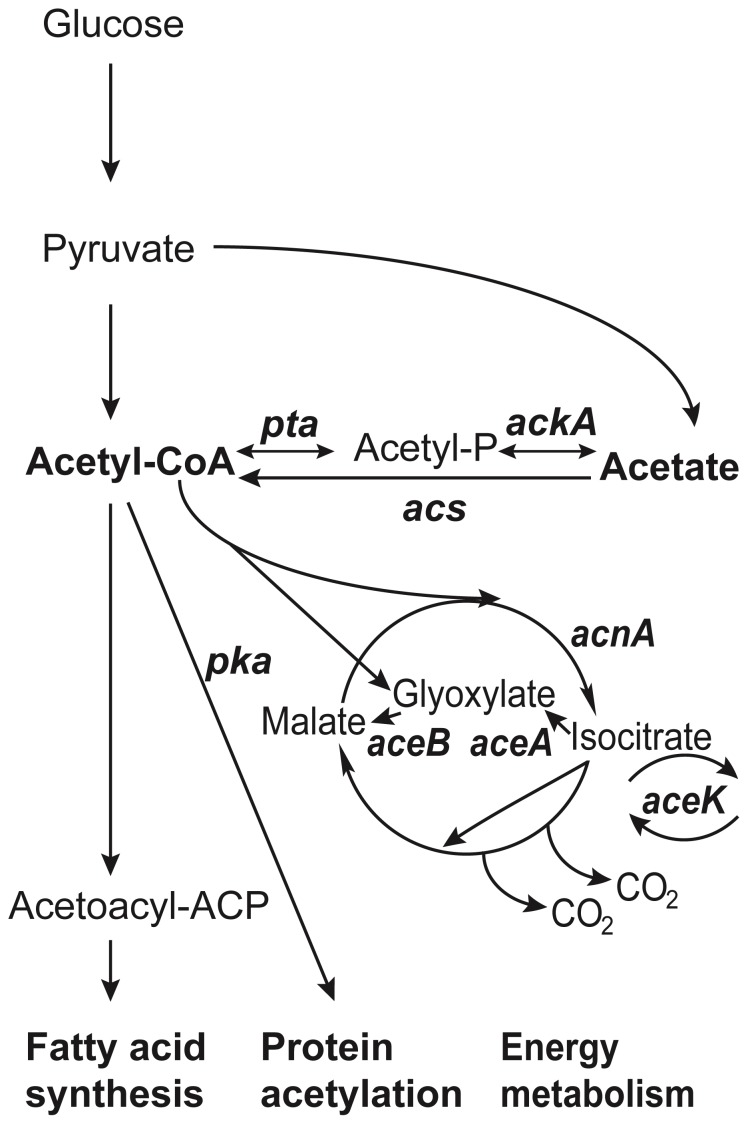
Outline of central metabolism indicating acetate production and utilization pathways. Outline of central metabolism showing the acetate synthesis and utilization pathways [24]. Gene names are shown in italics, substrates and products are referred to in the text.

**Figure 3:**
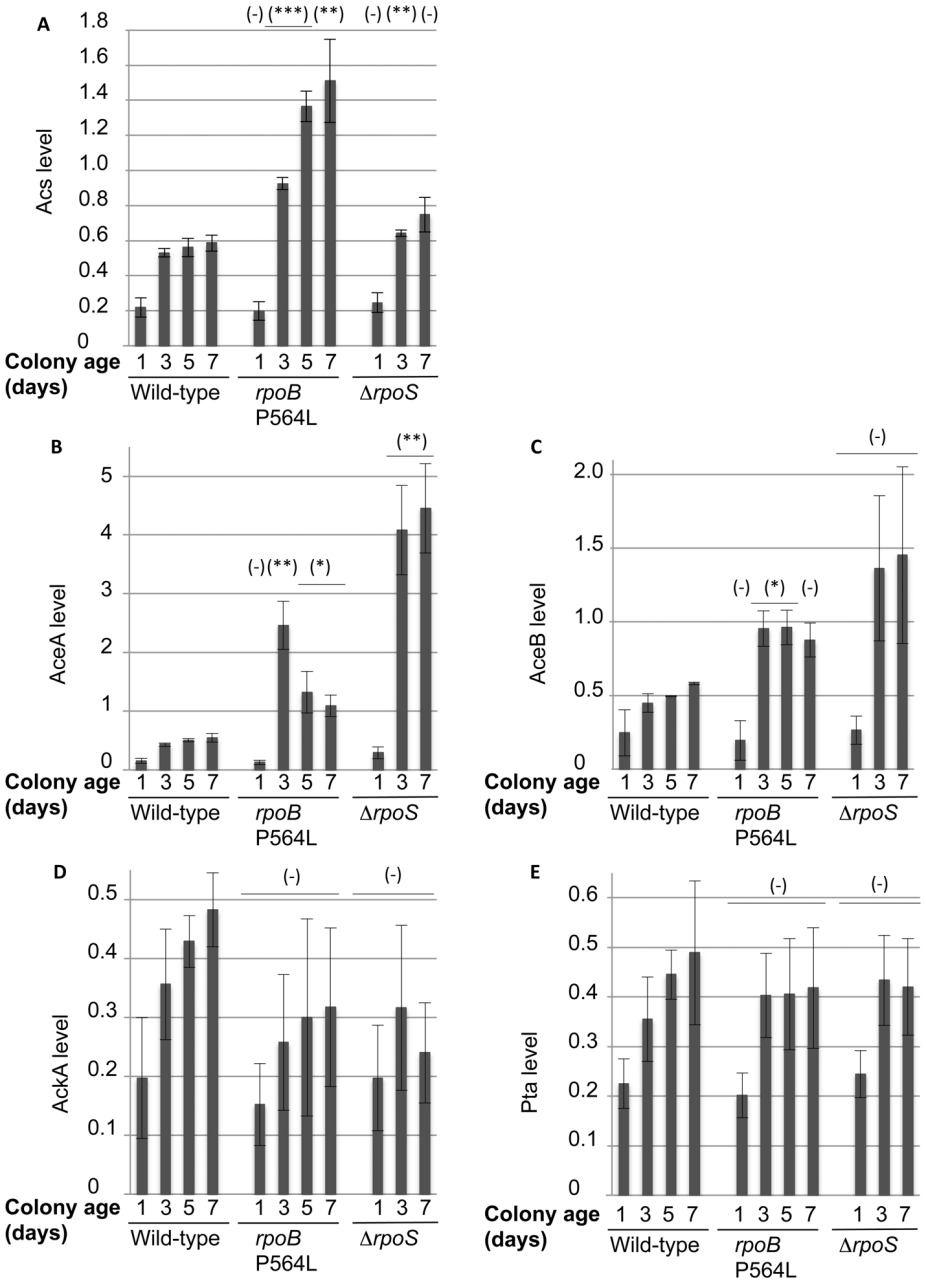
Protein quantification in aging colonies. Total protein was prepared from colonies of either wild-type, *rpoB* P564L mutant or Δ*rpoS* mutant, all grown for 1, 3, 5 and 7 days. Concentrations of the proteins of interest were quantified by Single Ion Monitoring mass spectrometry. Bars represent averages of two or three peptides per protein, measured in biological duplicates, each measured twice, with error bars representing standard deviation between the runs. Concentration values are in arbitrary units, normalized to total protein in the samples, to enable direct comparison between different days and different samples. The day 5 sample from the Δ*rpoS* mutant could not be analyzed. Statistical significance of differences, compared to wild-type samples of the same age, are indicated in the figure (-  =  not significant, *  = 95% confidence interval, **  = 99% conficence interval, ***  = 99.9% confidence interval). **A**. Concentration of Acs, acetyl-CoA synthase. **B**. Concentration of AceA, isocitrate lyase. **C**. Concentration of AceB, malate synthase. **D**. Concentration of AckA, acetate kinase. **E**. Concentration of Pta, phosphotransacetylase.

Single Ion Monitoring mass spectrometry was used to quantify seven proteins associated with acetate utilization in mutant and wild-type bacterial colonies aged for 1, 3, 5 or 7 days, using a protocol described by Thermo Fisher Scientific [20]. The proteins quantified were: ActP, acetate/glycolate permease; AckA, acetate kinase; Pta, phosphate acetyltransferase; Acs, acetyl-CoA synthetase; AceA, isocitrate lyase; AceB, malate synthase A; and Pka (PatZ, YfiQ), peptidyl-lysine acetyltransferase (Figure 2). At least two peptides per protein were analyzed and each sample was measured in biological replicates. The concentration of each of these proteins was quantified and compared between the wild-type and the *rpoB* and *rpoS* mutant strains (Figure 3). Acs (Figure 3A) was upregulated in all colonies as a function of colony age, dramatically so in the *rpoB* mutant strain. Due to high variation, the apparent increase in Acs levels for the 7 day-old *rpoS* mutant was not significantly different from the 7 day-old wild-type. The glyoxylate shunt enzymes, AceA and AceB, were strongly upregulated as a function of colony age in each of the mutant strains (Figure 3B and 3C). Also for AceB levels, the aged *rpoS* samples showed a high variation, which made the apparent increase, compared to the wild-type insignificant (Figure 3C). AckA and Pta levels increased after day 1 in all colony types but the levels did not differ significantly between wild-type and mutant strains (Figure 3D and 3E). The levels of Pka and ActP were too low in all samples to be reliably quantified. This quantitative proteome data confirmed that key enzymes required for the capture of acetate (by converting it to acetyl-CoA), and acetate utilization via the glyoxylate shunt, are present at significantly higher levels in stationary phase colonies, and that the levels of Acs, AceA, AceB are higher in the *rpoB* and *rpoS* mutant strains than in the wild-type (Figure 3, panels A, B, C). This correlation suggested that the activity of one or more of these enzymes might be required to support the continued growth of *rpoB* and *rpoS* mutants on aging wild-type colonies. This hypothesis was tested genetically.

### The ability to convert acetate to acetyl-CoA is important for subpopulation growth on aging wild-type colonies

We addressed whether the acetate that exponentially growing bacteria excrete might be important to support the growth of mutants in the stationary phase colony. Acetate can freely permeate the membrane [21], [22], [23] and the pathways of acetate metabolism have been thoroughly reviewed [24] and are outlined in Figure 2. To utilize acetate for protein acetylation or as a nutrient it must first be converted to acetyl-CoA. Conversion depends on either of two parallel pathways. The *acs* pathway (acetyl-CoA synthase) is a high affinity system used at low acetate concentrations whereas the constitutive *ackA-pta* pathway (acetate kinase, *ackA*, and phosphotransacetylase, *pta*) is used when acetate is available at relatively high concentrations [25]. Cells lacking both the *acs* and the *ackA-pta* pathways are unable to grow on acetate at any concentration [26].

**Figure 4:**
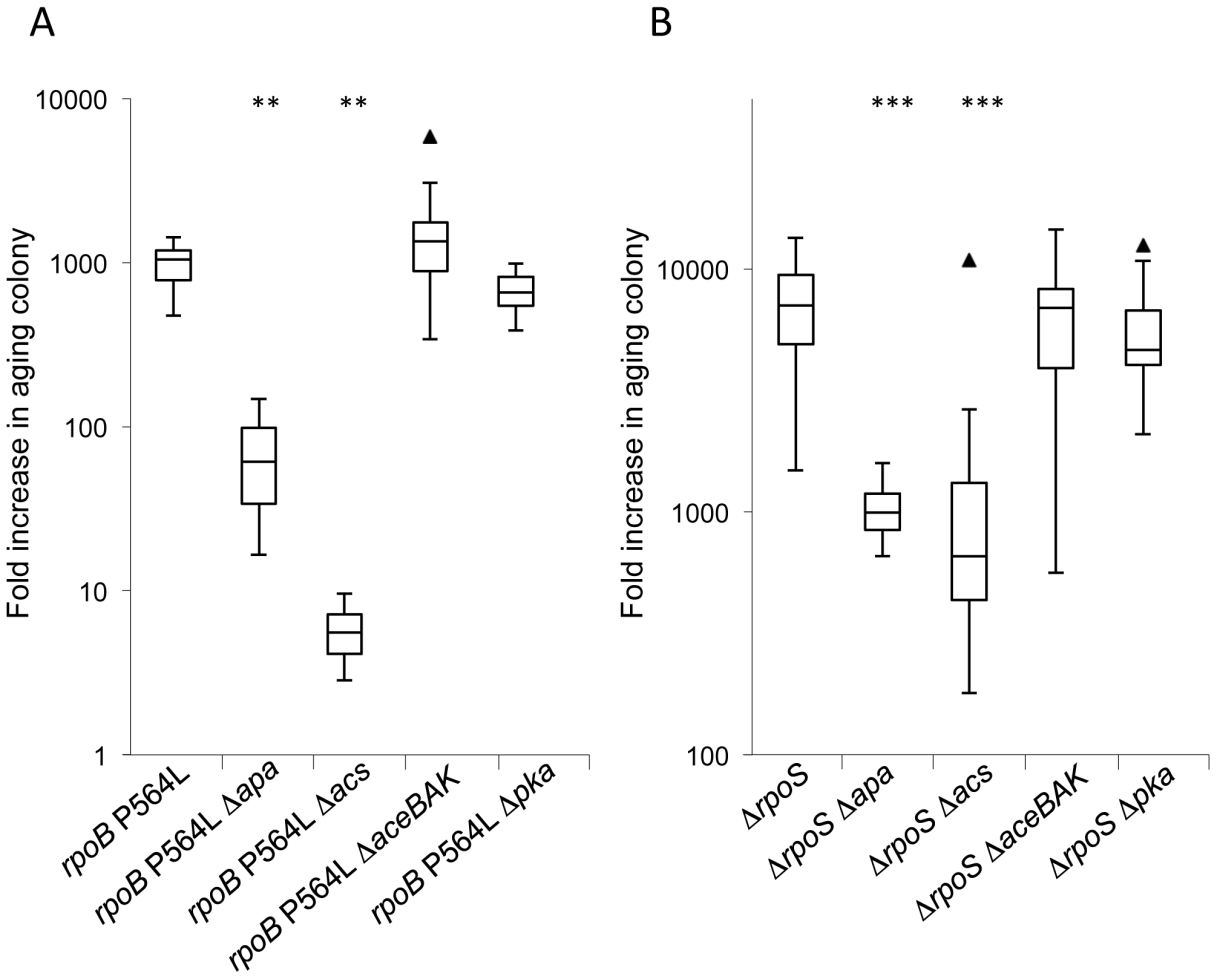
Influence of mutations affecting acetate metabolism on the growth advantage of *rpoB* P564L and Δ*rpoS* mutants. Fold increase in wild-type and mutant cells added to 24 h wild-type colonies and allowed to age for a further 7 days, as a function of acetate metabolism activity. **A**. Relative to the *rpoB* 564L mutant. **B**. Relative to the Δ*rpoS* mutant. The box plots show the first quartile, median, and third quartile values. Outliers are indicated as triangles. Δ*apa* indicates deletion of the three genes, *ackA-pta* and *acs*. Statistical significance of differences, compared to the *rpoB* or *rpoS* mutants, is given in Table S2 and is indicated in the figure by asterisks (**  = 99% confidence interval, ***  = 99.9% confidence interval).

Isogenic strains were constructed in which either *acs* alone or all three genes, *ackA, pta,* and *acs,* were deleted (labelled as Δ*apa* in Figure 4). These strains were otherwise wild-type, or carried either the *rpoB* P564L or Δ*rpoS* alleles. The ability of each of these strains to grow when added as a small population onto an aging wild-type colony was measured. Inactivation of the ability to convert acetate to acetyl-CoA reduced the growth advantage of each of the mutants but did not significantly affect the wild-type (Figure 4, Table S2). A significant reduction in mutant growth also occurred when only *acs* was knocked out. This shows that the ability to convert acetate to acetyl-CoA, dependent on an active *acs* gene, is crucial for these mutants subpopulations to grow and accumulate on aging wild-type colonies.

### The glyoxylate shunt is not required for *rpoB* and *rpoS* mutant growth on aging colonies

Growth on acetate as the sole carbon source requires the glyoxylate shunt, and thus the activities of the *aceB*, *aceA* and *aceK* gene products (Figure 2) to bypass the two CO_2_ evolving steps of the TCA cycle, at the expense of energy [27]. This bypass allows the production of four-carbon compounds such as malate and oxaloacetate during growth on two-carbon substrates like acetate. We constructed isogenic strains lacking the genes of the glyoxylate shunt. Deletion of *aceBAK* did not significantly reduce the ability of *rpoB* or *rpoS* mutants to grow on aging colonies (Figure 4 and Table S2). This shows that the growth of subpopulations of *rpoB* and Δ*rpoS* mutants on aging wild-type colonies does not depend on the activity of the glyoxylate shunt.

### Protein acetylation by Pka is not essential for mutant growth on aging colonies

Acetyl-CoA is also important as the source of donor acetyl groups for the acetylation of many proteins. Many of the enzymes involved in the central metabolic pathways of *E. coli* and *S.* Typhimurium are subject to lysine acetylation [28], [29], [30], [31], [32] and reversible acetylation has been shown to modulate enzyme activity [33], [34]. In *Salmonella*, protein actetylation is dependent on the peptidyl-lysine acetyltransferase encoded by *pka* (synonyms: *yfiQ*, *pat*) [35], [36], [37]. We asked whether Pka activity was essential for the ability of the *rpoB* or *rpoS* mutants to grow on aging wild-type colonies. Isogenic wild-type and mutant strains with the *pka* gene deleted were constructed and assayed for their ability to grow on aging wild-type colonies. Deletion of *pka* had no significant affect on the ability of any of the mutant strains to grow on the aging wild-type colonies (Figure 4, Table S2).

### Differential rate of acetate uptake and conversion in young and aged colonies

**Figure 5:**
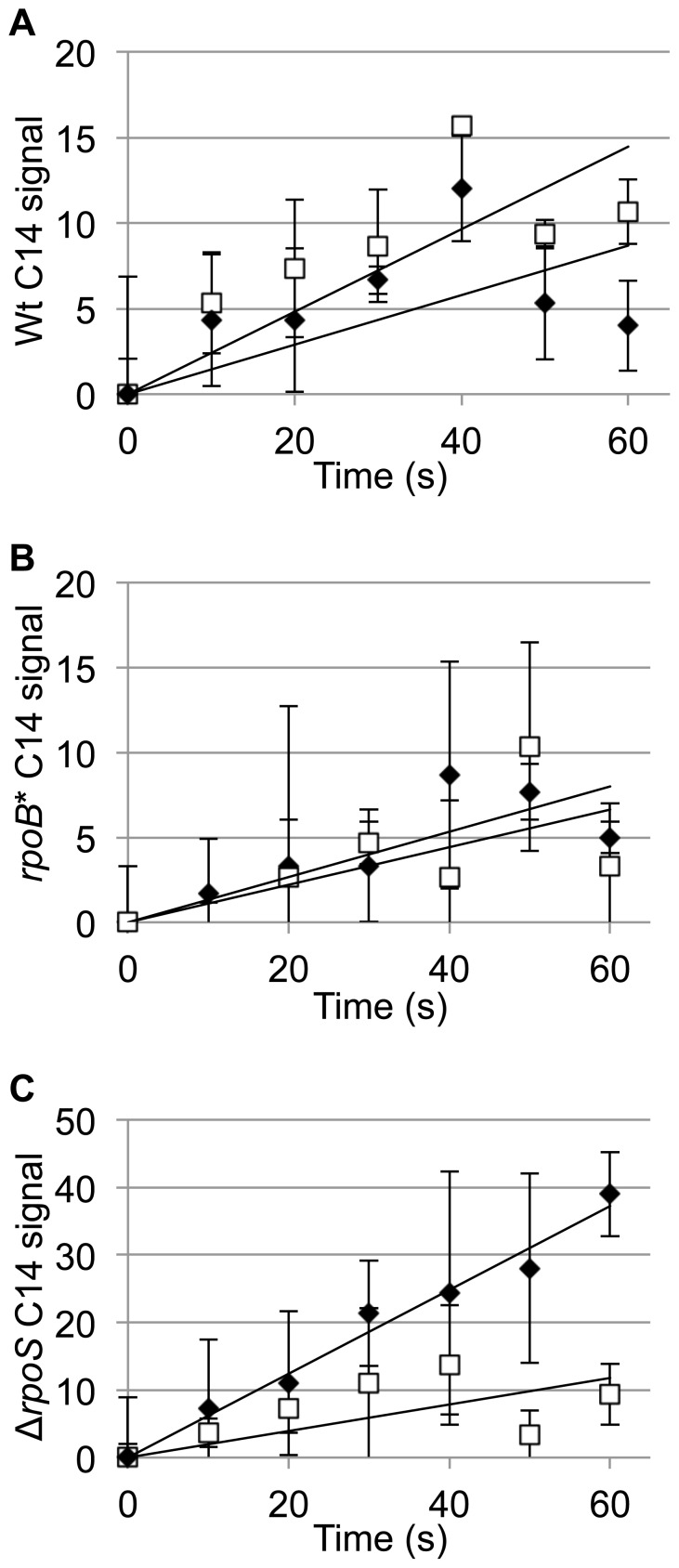
Acetate uptake rate in young and aged colonies. Colonies of 24 h (open squares) and 7 days (closed diamonds) were suspended in ^14^C-acetate. Samples were taken every 10 seconds for one minute. **A**. Wild-type colonies. **B**. *rpoB* P564L colonies. **C**. Δ*rpoS* colonies. Each data point represents the average of three independent measurements, with standard deviations as error bars.

The genetic tests showed that Acs, the enzyme that converts acetate to acetyl-CoA, is critically important for the ability of subpopulations of *rpoB* and *rpoS* mutants to continue growing as wild-type colonies age. Because acetate itself can freely diffuse across the cell membrane, the enzymatic action of Acs functions to capture acetate inside the bacterial cell where it can then be used for a variety of biological purposes (energy generation, fatty acid synthesis, protein acetylation). We compared acetate capture by wild-type and mutant cells in aging colonies that had been exposed to C^14^-acetate and measured the rate of the accumulation of C^14^ as a function of colony age. Colonies of wild-type, *rpoB* mutant and *rpoS* mutant were grown for either 24 h or 7 days. Colonies were suspended and washed in saline, C^14^-acetate was added to the suspensions and samples were taken every 10 seconds for 1 minute. In cells from 1 day-old colonies the rate of C^14^ uptake was similar for all strains (Figure 5). In contrast, in 7 day-old colonies, the rate of uptake was reduced in the wild-type, it was unchanged in the *rpoB* mutant, and it increased almost 5-fold in the *rpoS* mutant (Figure 5).

### The growth advantage of subpopulations is increased on aging colonies that are unable to convert acetate to acetyl-CoA

**Figure 6:**
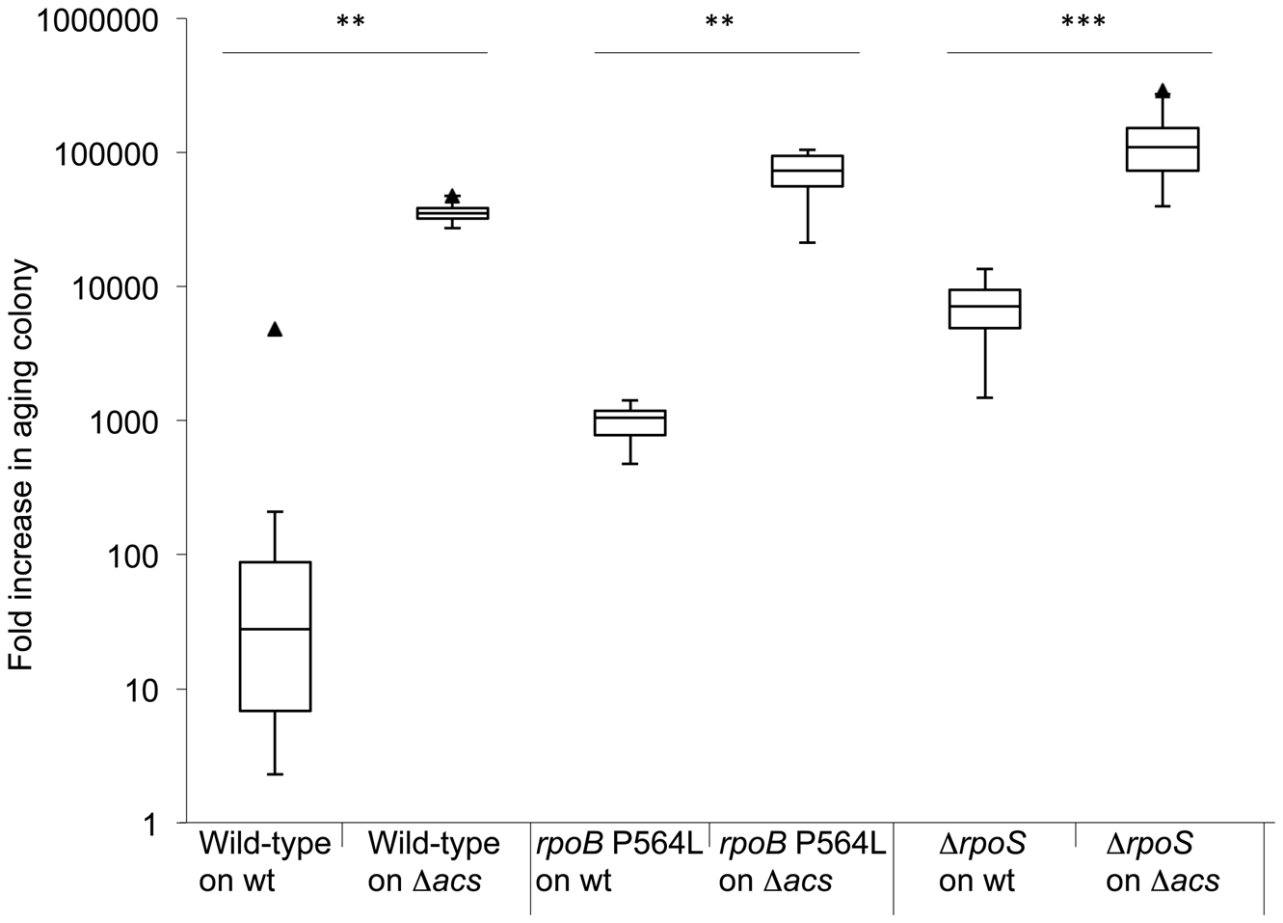
Influence of the *acs* status of the background colony on the growth advantage of Rif^R^ and RpoS mutants. Fold increase in wild-type and mutant cells added to 24 h wild-type or Δ*acs* colonies and allowed to age for a further 7 days. The box plots show the first quartile, median, and third quartile values. Outliers are indicated as triangles. Statistical significance of differences between strains is given in Table S2 and is indicated in the figure by asterisks (**  = 99% confidence interval, ***  = 99.9% confidence interval).

Bacteria in a wild-type colony excrete acetate during exponential growth, and then import and consume acetate during the transition into stationary phase. We hypothesized that if cells in the aging wild-type colony were unable to consume that acetate during its transition into stationary phase, that more acetate would then be available to support the growth of subpopulations of mutant cells. We tested this hypothesis by measuring the accumulation of isogenic wild-type and *rpoB* and *rpoS* mutants on aging colonies of a Δ*acs* mutant. The prediction was that the subpopulations of added cells would accumulate to greater numbers than when added onto aging wild-type colonies, because there should be a greater quantity of acetate available in aging colonies of a Δ*acs* mutant to support their growth. This prediction was borne out by the experiment (Figure 6, Table S4). All added subpopulations grew to a significantly greater number than when placed on aging wild-type colonies. Interestingly, the added wild-type subpopulation also showed enhanced accumulation on the Δ*acs* mutant. This data show that the relative availability of acetate is a critical factor, both enabling and limiting the growth of *rpoB* and *rpoS* mutant subpopulations on aging wild-type colonies.

### Summary and conclusions

We have shown that mutations affecting RNA polymerase (Δ*rpoS* and *rpoB* P564L) facilitate continued growth of mutant cells on aging colonies of wild-type *S.* Typhimurium after most cells have entered stationary phase. We have also shown that the continued growth of these mutant subpopulations on aging colonies depends to a large extent on the availability of acetate and on the capacity of the mutant cells to import and convert that acetate into acetyl-CoA. We suggest from these data that the RNA polymerase status of individual bacteria in a population, in association with acetate availability, exerts a strong influence in determining whether *S.* Typhimurium enters stationary phase or continues to grow as the colony ages.

We have shown that the conversion of acetate to acetyl-CoA is important for the bacteria to be able to grow in the aging colony. However, since neither the glyoxylate shunt nor protein acetylation is required for growth, this suggests that the generated acetyl-CoA may be used for something else. Apart from the TCA cycle and protein acetylation, acetyl-CoA is also used in fatty acid synthesis [38]. It is tempting to speculate that the acetyl-CoA in the aging colonies may be utilized to synthesize fatty acids.

The phenomenon of continued growth of mutant sub-populations in stationary phase colonies (or liquid cultures) could have a general evolutionary significance. It illustrates the temporal and environmental dependency of the concept of relative fitness, and the interplay between spontaneous mutations, changing environmental selection pressures, and sibling rivalry, within clonal populations.

## Materials and Methods

### Bacterial strains and growth conditions

*S. enterica* serovar Typhimurium ATTC strain 14028 s for which the complete genome sequence is available [39] was used as wild-type and isogenic strains were constructed from it. Transductions were made using phage P22 HT [40]. Deletions were made by λ-red recombineering [10], [11] replacing the relevant genes with an FRT-*tetRA*-FRT cassette as described previously [41]. The genotypes of the bacterial strains used are listed in Table 1. Oligonucleotides used for recombineering and/or DNA sequencing across deletion junctions are listed (Table S5). Drug resistance markers were removed from recombineered strains by site-specific recombination after transduction with a P22 phage lysate grown on a strain carrying the plasmid pCP20 (Amp^R^) expressing Flp recombinase [42], leaving one FRT sequence at the site of deletion. Bacteria were grown at 37°C in Luria-Bertani broth (LB) or on Luria Agar, LA (LB supplemented with 1.5% agar; Oxoid, Basingstoke, England; 0.2% glucose; 3 mM CaCl_2_). Antibiotics were used at the following final concentrations; tetracycline 15 µg/mL, ampicillin 100 µg/mL, rifampicin 100 µg/mL. For colony-aging experiments agar plates were incubated at 37°C in sealed plastic bags to minimize dehydration.

**Table 1:** Strain list.

Strains^a^	Genotype
TH6509	14028s Wild-type
TH6694	*zhe-8953*::Tn*10*dTe*t*
TH7705	Δ*acs*
TH7141	*rpoB* P564L
TH7148	*zhe-8953*::Tn*10*dTet *rpoB* P564L
TH7718	*zhe-8953*::Tn*10*dTet Δ*aceBAK rpoB* P564L
TH8168	*zhe-8953*::Tn*10*dTet Δ*ackA-pta* Δ*acs rpoB* P564L
TH7722	*zhe-8953*::Tn*10*dTet Δ*acs rpoB* P564L
TH8486	*zhe-8953*::Tn*10*dTet Δ*pka rpoB* P564L
TH8082	*ΔrpoS*
TH8097	*zhe-8953*::Tn*10*dTet Δ*rpoS*
TH8163	*zhe-8953*::Tn*10*dTet Δ*aceBAK* Δ*rpoS*
TH8164	*zhe-8953*::Tn*10*dTet Δ*ackA-pta* Δ*acs* Δ*rpoS*
TH8516	*zhe-8953*::Tn*10*dTet Δ*acs* Δ*rpoS*
TH8487	*zhe-8953*::*Tn10*dTet Δ*pka* Δ*rpoS*

a All strains are isogenic to TH6509 *Salmonella enterica* serovar Typhimurium strain 14028 s.

### PCR and DNA sequencing

The public genome sequences for *S. enterica* LT2 (NCBI accession number NC_003197) and *S. enterica* 14028 s (GenBank accession number CP001363) were used to design primers for PCR amplification and DNA sequencing. DNA samples for PCR were obtained by dispersing fresh bacterial colonies in 100 µl of sterile water, heating at 95°C for 5 min and then cooling on ice. PCR was performed using PuReTaq Ready-To-Go PCR beads (GE Healthcare, Uppsala, Sweden) according to the protocol of the manufacturer. Amplifications were carried out in 25 µl volumes containing 0.4 µM reverse and forward primers and 1 µl of DNA sample using a DNA engine PTC-200 thermocycler (SDS-diagnostics, Falkenberg, Sweden). PCR was initiated by denaturation at 95°C for 5 min, followed by 25 cycles of 95°C for 30 s, 56°C for 20 s, and 72°C for 1 min. Amplification products were visualized by agarose gel electrophoresis and ethidium bromide staining to assess the sizes of the gene fragments. Products were purified prior to sequencing using the QIAquick PCR purification kit (Qiagen, VWR International AB, Stockholm, Sweden) according to the manufacturer's instructions. PCR product concentrations were quantified using a Nanodrop NO-1000 spectrophotometer (Nanodrop, Wilmington, DE, USA). DNA sequencing of purified PCR products was performed at Macrogen Inc., Seoul, South Korea.

### Assay of mutant growth on aging wild-type colonies

A fresh overnight culture was diluted in 0.9% NaCl and a 2 µl volume containing 100–1000 cfu was spotted onto an 82 mm diameter 0.2 µm pore size Protran BA83 nitrocellulose filter (Whatman, Dassel, Germany) on LA plates, to initiate growth of a wild-type colony. After 24 h incubation a 4 µl volume containing approximately 2×10^3^ cfu of wild-type or an isogenic mutant, each marked with the same tetracycline resistance marker, *zhe*-8953::Tn*10*dTet, was spotted onto the 24 h colony. After this addition the colonies were incubated for an additional 7 days at 37°C in sealed plastic bags (the period of colony aging) after which the colony was suspended in 1 ml of 0.9% NaCl and appropriate dilutions were plated on LA and LA-Tet plates. The median fold increase in the number of mutant Tet^R^ cells over the 7-day aging period was calculated. Each colony aging competition experiment was biologically independent: separate independent cultures of wild-type bacteria were grown to initiate each independent colony; separate independent cultures of Tet^R^-marked wild-type or mutant cells were grown for addition to each independent 24 h colony. Under these incubation conditions a bacterial cell undergoes up to 30 generations of growth and division within 24 h, reaching a population of approximately 10^9^ cells in the colony [1], [2], [4]. Prior to 24 h the rate of growth slows significantly and the population initiates the process of entering the stationary phase. When incubation is continued beyond 24 h the population in the colony continues to grow but at a much slower rate, reaching approximately 10^10^ by 48–72 h. No significant further net increase in population size occurs if the incubation is continued longer [1], [2], [4].

### Protein preparation and mass spectrometry measurements

Colonies of pure wild-type, pure *rpoB* P564L mutant or pure Δ*rpoS* mutant were initiated and aged. After the appropriate time, the colonies were cut out and suspended in 1 ml 0.9% NaCl. The cells were pelleted and lysed by the addition of 400 µl lysis buffer (20 mM HEPES pH 8.0, 9 M urea) containing a protease inhibitor cocktail added according to the manufacturers instructions (Complete, Mini, EDTA-free, Roche Diagnostics Scandinavia AB, Bromma, Sweden) and mixed by pipetting. The samples were sonicated with a 16 micron probe for 3×20 seconds, and cooled on ice 1 minute between each sonication. The lysates were centrifuged at 12000 g for 15 min at 4°C. Shotgun Proteome Analysis, and Quantitative Single Ion Monitoring were each carried out at the Science for Life Mass Spectrometry Technology Platform, Uppsala University, Sweden. An LTQ-Orbitrap Velos Pro ETD mass spectrometer (Thermo Fisher Scientific) was used for the mass spectrometry measurements and the PinPoint 1.3 software was used for the quantitation analysis [20].

### Uptake of ^14^C-acetate

Colonies were initiated as described for the assay for the aged colonies, except that these colonies were pure wild-type, pure *rpoB* P564L or pure Δ*rpoS*. After the appropriate number of days of incubation at 37°C, the colonies were cut out and suspended in 10 ml 0.9% NaCl at room temperature. Immediately prior to initiating the assay a time zero sample was taken by transferring 750 µl cell suspension to 750 µl non-radioactive 2 mM sodium acetate at room temperature. To initiate the assay 10 µl (0.002 µCi) ^14^C-acetate (Perkin Elmer, Upplands Väsby, Sweden, 50–62 mCi/mmol) was added to the cell suspension. Samples were then taken every 10 seconds for 1 minute by the removal of 750 µl cell suspension to 750 µl non-radioactive 2 mM sodium acetate chase solution at room temperature. The samples were chased for 8 minutes and then placed on ice. Cells were pelleted by centrifugation (1 min, 12000×g, 4°C) and washed three times in 500 µl 0.9% NaCl. The last pellet was re-suspended in 200 µl 0.9% NaCl and added to 4 ml scintillation fluid (Quicksafe Flow 2, Zinsser Analytic), vortexed, then placed in a scintillation counter. The uptake of ^14^C-acetate in bacteria was analyzed by plotting radioactive incorporation as a function of time. The slope of the linear uptake curve was considered to be the specific acetate uptake ability of the strain.

### Statistical analysis

The significance of the differences in fold increase of mutant subpopulations between different isogenic strains was calculated using the Mann-Whitney nonparametric test (http://vassarstats.net). The significance of differences in acetate uptake and in protein quantity was calculated by unpaired two-tailed t-tests (http://graphpad.com/quickcalcs/ttest1/).

## Supporting Information

Table S1 **High frequency of** ***rpoS*** **mutations among extremely aged colonies of** ***S. enterica*** **LT2.** TH7792 *S. enterica* LT2 (*zfd-6825*::Tn*10*) colonies were aged for 15 weeks at 37°C, when surviving cells were examined for evidence of mutations affecting RNA polymerase. The *rpoA, rpoB, rpoC, and rpoS* genes from 18 independent clones were sequenced. No mutations were identified in *rpoA, rpoB*, or *rpoC*. However, in three of the 18 strains, mutations were identified in *rpoS*. The Tet^R^ phenotype had been lost in 5/18 strains but two of the RpoS mutants had retained Tet^R^ and were tested for growth advantage in a standard colony aging experiment. Each RpoS mutant had a growth advantage relative to the isogenic wild-type in the aging colony. Thus, under extreme aging conditions a significant fraction (3/18) of the surviving bacteria had acquired mutations in *rpoS*, and had also acquired a growth advantage in aging colonies.
(DOCX)

Table S2 **Inactivation of acetate utilization genes reduces the growth of** ***rpoS*** **and** ***rpoB*** **mutant subpopulations on aging wild-type^a^ colonies.** Colony competition experiments were made as described in Materials and Methods, using isogenic strains carrying the mutations listed.
(DOCX)

Table S3 **Proteomic analysis of wild-type and mutant strains (** ***rpoB*** **P564L, and Δ** ***rpoS*** **).** The data show individual relative protein levels in Day 1 and Day 7 colonies, and the fold change over that time period.
(XLSX)

Table S4 **Inactivation of the** ***acs*** **gene in the background colony increases the growth of wild-type,** ***rpoB*** **and** ***rpoS*** **mutant subpopulations on aging colonies, compared to aging on wild-type background colonies.** Colony competition experiments were made as described in Materials and Methods except that the background colony had the genotype Δ*acs*.
(DOCX)

Table S5 **Oligonucleotides used to make and confirm genetic deletions.** List of oligonucleotides used in recombineering mutations into the chromosome, for PCR, and as primers for DNA sequencing.
(DOCX)
