# Supplementary material for: Acetate Availability and Utilization Supports the Growth of Mutant Sub-Populations on Aging Bacterial Colonies
Source: PLoS One. 2014 Oct 2;9(10):e109255. doi: 10.1371/journal.pone.0109255 (PMC4183559; doi:10.1371/journal.pone.0109255)
Supplement: Table S1 — High frequency of rpoS mutations among extremely aged colonies of S. enterica LT2. TH7792 S. enterica LT2 (zfd-6825::Tn10) colonies were aged for 15 weeks at 37°C, when surviving cells were examined for evidence of mutations affecting RNA polymerase. The rpoA, rpoB, rpoC, and rpoS genes from 18 independent clones were sequenced. No mutations were identified in rpoA, rpoB, or rpoC. However, in three of the 18 strains, mutations were identified in rpoS. The TetR phenotype had been lost in 5/18 strains but two of the RpoS mutants had retained TetR and were tested for growth advantage in a standard colony aging experiment. Each RpoS mutant had a growth advantage relative to the isogenic wild-type in the aging colony. Thus, under extreme aging conditions a significant fraction (3/18) of the surviving bacteria had acquired mutations in rpoS, and had also acquired a growth advantage in aging colonies. (DOCX) [file pone.0109255.s001.docx]

**Table S1**

**Mutations selected in LT2 *rpoS* (3/18 random clones) after extended colony aging^1^**

| **Strain** | ***rpoA, B, C*** | **Mutations in *rpoS* sequence** | **C.I.^2^** |
| --- | --- | --- | --- |
| TH7792^3^ | W.T. | W.T. | 1 |
| TH7793 | W.T. | Ser90Phe (TCT→TTT) | 237 |
| TH7794 | W.T. | Deletion 75 bp (nt 677-751) | n.d.^4^ |
| TH7795 | W.T. | Trp148Stop (TGG→TAG) | 579 |

^1^ Colonies were incubated at 37°C for 15 weeks in sealed plastic bags.

^2^ C.I. Competitive index on aging TH4527.

^3^ The competitive index (C.I.) of TH7792 is set to 1 and all other C.I. values are expressed relative this. The C.I. is expressed as a median value based on 4 independent assays.

^4^ n.d. Not determined.
