# Supplementary material for: Acetate Availability and Utilization Supports the Growth of Mutant Sub-Populations on Aging Bacterial Colonies
Source: PLoS One. 2014 Oct 2;9(10):e109255. doi: 10.1371/journal.pone.0109255 (PMC4183559; doi:10.1371/journal.pone.0109255)
Supplement: Table S2 — Inactivation of acetate utilization genes reduces the growth of rpoS and rpoB mutant subpopulations on aging wild-typea colonies. Colony competition experiments were made as described in Materials and Methods, using isogenic strains carrying the mutations listed. (DOCX) [file pone.0109255.s002.docx]

**Table S2.**

| Strain | Genotype^b^ | Fold increase^c^ | N^d^ | P values (two tailed)^e^ | | |
| --- | --- | --- | --- | --- | --- | --- |
|  |  |  |  | wt | *rpoB* | Δ*rpoS* |
| TH6694 | wild-type | 28 | 11 | - | **0.0034** | **<0.0001** |
| TH7148 | *rpoB* P564L | 1046 | 8 | **0.0034** | - | n.r. |
| TH8097 | Δ*rpoS* | 7078 | 24 | **<0.0001** | n.r. | - |
| TH7722 | Δ*acs rpoB* P564L | 6 | 9 | n.r. | **0.0006** | n.r. |
| TH8516 | Δ*acs* Δ*rpoS* | 656 | 15 | n.r. | n.r. | **<0.0001** |
| TH8168 | Δ*ackA-pta* Δ*acs rpoB* P564L | 61 | 8 | n.r. | **0.0009** | n.r. |
| TH8164 | Δ*ackA-pta* Δ*acs* Δ*rpoS* | 997 | 8 | n.r. | n.r. | **<0.0001** |
| TH7718 | Δ*aceBAK rpoB* P564L | 1350 | 19 | n.r. | 0.1770 | n.r. |
| TH8163 | Δ*aceBAK* Δ*rpoS* | 6914 | 19 | n.r. | n.r. | 0.6312 |
| TH8486 | Δ*pat rpoB* P564L | 658 | 8 | n.r. | 0.0658 | n.r. |
| TH8487 | Δ*pat* Δ*rpoS* | 4629 | 24 | n.r. | n.r. | 0.0969 |

**Inactivation of acetate utilization genes reduces the growth of *rpoS* and *rpoB* mutant subpopulations on aging wild-type^a^ colonies**

^a^ The aging wild-type colony (onto which subpopulations were added) is TH6509. *S. enterica* 14028s.

^b^ In addition to the mutations indicated, all subpopulation strains added at 24 h carried *zhe*-8953::Tn*10*dTet as a phenotypic marker.

^c^ Median fold increase from the time of addition (24 h) until 7 additional days had elapsed relative to the wild-type TH6694. The wild-type strain TH6694 increased 28-fold (median value) equivalent to 4-5 cell doublings during the 7 day aging period, compared to approximately 30 cell doublings during the initial 24 h growth period.

^d^ N is number of independent aging experiments. Independent cultures were used to initiate each wild-type colony, and to initiate each genetically marked strain added at 24 h.

^e^ Two-tailed P-values (Mann-Whitney test). Values significant at the 95% confidence level are shown in bold. P-values were calculated relative to wild-type (TH6694), the *rpoB* P564L mutant (TH6879) and the Δ*rpoS* mutant (TH8097).
