# Supplementary material for: Acetate Availability and Utilization Supports the Growth of Mutant Sub-Populations on Aging Bacterial Colonies
Source: PLoS One. 2014 Oct 2;9(10):e109255. doi: 10.1371/journal.pone.0109255 (PMC4183559; doi:10.1371/journal.pone.0109255)
Supplement: Table S4 — Inactivation of the acs gene in the background colony increases the growth of wild-type, rpoB and rpoS mutant subpopulations on aging colonies, compared to aging on wild-type background colonies. Colony competition experiments were made as described in Materials and Methods except that the background colony had the genotype Δacs. (DOCX) [file pone.0109255.s004.docx]

**Table S4**

| Strain | Genotype^b^ | Fold increase^c^ | N^d^ | P values (two tailed)^e^ | | |
| --- | --- | --- | --- | --- | --- | --- |
|  |  |  |  | wt/wt | *rpoB*/wt | Δ*rpoS*/wt |
| TH6694 | wild-type | 35089 | 8 | **0.0003** | **-** | **-** |
| TH7148 | *rpoB* P564L | 73199 | 8 | **-** | **0.0009** | - |
| TH8097 | Δ*rpoS* | 110463 | 8 | **-** | - | **<0.0001** |

**Inactivation of the *acs* gene in the background colony^a^ increases the growth of wild-type, *rpoB* and *rpoS* mutant subpopulations on aging colonies, compared to aging on wild-type background colonies.**

^a^ The aging Δ*acs* colony (onto which subpopulations were added) is TH7705. *S. enterica* 14028s.

^b^ In addition to the mutations indicated, all subpopulation strains added at 24 h carried *zhe*-8953::Tn*10*dTet as a phenotypic marker.

^c^ Median fold increase from the time of addition (24 h) until 7 additional days had elapsed.

^d^ N is number of independent aging experiments. Independent cultures were used to initiate each wild-type colony, and to initiate each genetically marked strain added at 24 h.

^e^ Two-tailed P-values (Mann-Whitney test). Values significant at the 95% confidence level are shown in bold. P-values were calculated relative to wild-type (TH6694), the *rpoB* P564L mutant (TH6879) and the Δ*rpoS* mutant (TH8097), aged on wild-type background colonies (TH6409).
