# Supplementary material for: Acetate Availability and Utilization Supports the Growth of Mutant Sub-Populations on Aging Bacterial Colonies
Source: PLoS One. 2014 Oct 2;9(10):e109255. doi: 10.1371/journal.pone.0109255 (PMC4183559; doi:10.1371/journal.pone.0109255)
Supplement: Table S5 — Oligonucleotides used to make and confirm genetic deletions. List of oligonucleotides used in recombineering mutations into the chromosome, for PCR, and as primers for DNA sequencing. (DOCX) [file pone.0109255.s005.docx]

**Table S5**

**Oligonucleotides used to make and confirm genetic deletions.**

| Name | Sequence 5’ – 3’ |
| --- | --- |
| aceB1^a^ | ATGAATCCACAGGCAACCACAACTGATGAATTAACCTTTATGAAGTTCCTATACTTTCTAGAGAATAGGAACTTCCACCAAACACCCCCCAAAACC |
| aceB2^b^ | TTAAGCCAGTAAGCGATAGCCCGGCAGGGTGAGGAAGTCGATGAAGTTCCTATTCTCTAGAAAGTATAGGAACTTCACACACAACCACACCACACCAC |
| AceB3 | cctaaagcgtttcagcatgt  Forward primer to confirm knockout of *aceB*, upstream of *aceB* |
| AceB4 | cagcgcggttgtgtccactctt  Reverse primer to confirm knockout of *aceB*, downstream of *aceB* |
| aceA1^a^ | ATGAAAACCCGTACTCAACAAATCGAAGAATTACAGAAAGTGAAGTTCCTATACTTTCTAGAGAATAGGAACTTCCACCAAACACCCCCCAAAACC |
| aceA2^b^ | TCAAAACTGCGCTTCTTCGGTGGAACCCGTTAACGCGGTAACGAAGTTCCTATTCTCTAGAAAGTATAGGAACTTCACACACAACCACACCACACCAC |
| AceA3 | ggaagagatgcgggtaatcc  Forward primer to confirm knockout of *aceA*, upstream of *aceA* |
| AceA4 | gcaatcagtaattccaggcc  Reverse primer to confirm knockout of *aceA*, downstream of *aceA* |
| aceK1^a^ | ATGCCGCGTGGCCTGGAATTACTGATTGCTCAAACCATCCTGAAGTTCCTATACTTTCTAGAGAATAGGAACTTCCACCAAACACCCCCCAAAACC |
| aceK2^b^ | TTACGAAGAGTTCGCCGTACTTGAGATCGCGCCGTAACGCGAAGTTCCTATTCTCTAGAAAGTATAGGAACTTCACACACAACCACACCACACCAC |
| AceK3 | ccagcaggaagtgggtactg  Forward primer to confirm knockout of *aceK*, upstream of *aceK* |
| AceK4 | ggtcatatcacattgtgctcaac  Reverse primer to confirm knockout of *aceK*, downstream of *aceK* |
| acs1^a^ | ATGAGCCAAACACATAAACACGCCATTCCCGCCAACATTGTGAAGTTCCTATACTTTCTAGAGAATAGGAACTTCCACCAAACACCCCCCAAAACC |
| acs2^b^ | TTATGACGGCATCGCGATGGCCTGCTTCTCTTCGAGCAGTTTGAAGTTCCTATTCTCTAGAAAGTATAGGAACTTCACACACAACCACACCACACCAC |
| acs3 | CATTGATCTCCTCTATGTG  Forward primer to confirm knockout of *acs*, upstream of *acs* |
| acs4 | GTAACACGAAATCCTTTGGG  Reverse primer to confirm knockout of *acs*, downstream of *acs* |
| pta1^a^ | GTGTCCCGTATTATTATGCTGATCCCTACCGGAACCAGCGTGAAGTTCCTATACTTTCTAGAGAATAGGAACTTCCACCAAACACCCCCCAAAACC |
| pta2^b^ | TTACTGCTGCTGCTGAGAAGCCTGGATCGCCGTCAGGGCGATGAAGTTCCTATTCTCTAGAAAGTATAGGAACTTCACACACAACCACACCACACCAC |
| pta3 | TGCTGTTTTGTAACCCGTCC  Forward primer to confirm knockout of *pta*, upstream of *pta* |
| pta4 | GCCTACAACGGTATCTCACC  Reverse primer to confirm knockout of *pta*, downstream of *pta* |
| ackA1^a^ | ATGTCGAGTAAGTTAGTACTGGTTCTGAACTGCGGTAGTTTGAAGTTCCTATACTTTCTAGAGAATAGGAACTTCCACCAAACACCCCCCAAAACC |
| ackA2^b^ | TCAGGCAGTCAGACGGCTCGCGTCTTGCGCGATAACCAGTTCGAAGTTCCTATTCTCTAGAAAGTATAGGAACTTCACACACAACCACACCACACCAC |
| ackA3 | CGCTCTATGGCTCACTGACG  Forward primer to confirm knockout of *ackA*, upstream of *ackA* |
| ackA4 | CGTTCCATAGCACGGATGACG  Reverse primer to confirm knockout of *ackA*, downstream of *ackA* |
| pka1^a^ | TTTAAAATTATCCGGTCACTTCTGTGTAAGGGAAACCGGTTGAAGTTCCTATACTTTCTAGAGAATAGGAACTTCCACCAAACACCCCCCAAAACC |
| pka2^b^ | TCAGTACCCGTTAAAGTGGTCAACATTTCCAGTACATTACGAAGTTCCTATTCTCTAGAAAGTATAGGAACTTCACACACAACCACACCACACCAC |
| pka3 | GAACATTTCACCCGTTTCAA  Forward primer to confirm knockout of *pka*, upstream of *pka* |
| pka4 | AGAAATCTTAGGGAGTTGGG  Reverse primer to confirm knockout of *pka*, downstream of *pka* |
| rpoSFRT1^a^ | AAGGCCAGTCGACAGACTGGCCTTTTTTTGACAAGGGTACTGAAGTTCCTATACTTTCTAGAGAATAGGAACTTCCACCAAACACCCCCCAAAACC |
| rpoSFRT2^b^ | TTGCTAGTTCCGTCAAGGGATCACGGGTAGGAGCCAGGTTGAAGTTCCTATTCTCTAGAAAGTATAGGAACTTCACACACAACCACACCACACCAC |
| RpoS5426F | ctgctggcagaagacaaacg |
| RpoS6290R | ggtattcaccactgttaacagc |

^a^ These oligonucleotides are structured to generate in-frame deletions after Flp treatment: nts 1-40 are the specified gene coding sequence homology block (forward) from and including the start codon; nts 41-75 are the FRT sequence; nts 76-96 are a universal linker sequence UL1 to facilitate PCR amplification of a drug resistance cassette from TH6768.

^b^ These oligonucleotides are structured to generate an in-frame deletion after Flp treatment: nts 1-40 are the specified gene coding sequence homology block (reverse complement) from and including the translation termination codon; nts 41-75 are the FRT sequence; nts 76-96 are a universal linker sequence UR1 to facilitate PCR amplification of a drug resistance cassette from TH6768.

**Strain TH6768 used as source of tetracycline-resistance genes.**

TH6768. Originally from John Roth, TT25401 *S. enterica* (LT2) [*zfa-9223*::kan**zfa-9228*::*tetRA**Peut] *eut-38*::MudA. Comment: TR10000 background; swap of UNI-*tetRA* into kanS2, restoring kan and leaving *eut* control region intact; used as a source of UNI-flanked *tetRA* template for linear transformation.
